# Protein crystal lattices are dynamic assemblies: the role of conformational entropy in the protein condensed phase

**DOI:** 10.1107/S2052252517017833

**Published:** 2018-01-10

**Authors:** Margarita Dimova, Yancho D. Devedjiev

**Affiliations:** a Department of Anesthesiology, University of Virginia, 1215 Lee Street, Charlottesville, VA 22908, USA

**Keywords:** protein crystals, static disorder, dynamic disorder, conformational entropy, elastic molecular shape, oscillating crystal lattice, local entropic force, X-ray crystallography, crystallization, crystal growth

## Abstract

Until recently, the occurrence of conformational entropy in protein crystal contacts was considered to be a very unlikely event. A study based on the most accurately refined protein structures demonstrated that side-chain conformational entropy and static disorder might be common in protein crystal lattices. The present investigation uses structures refined using ensemble refinement to show that although paradoxical, conformational entropy is likely to be the major factor in the emergence and integrity of the protein condensed phase. This study reveals that the role of shape entropy and local entropic forces expands beyond the onset of crystallization. For the first time, the complete pattern of intermolecular interactions by protein atoms in crystal lattices is presented, which shows that van der Waals interactions dominate in crystal formation.

## Abstract 2

New insight into the nature of protein crystal lattices is proposed.

## 1. Introduction   

Baldwin analyzed the nature of the driving force in protein folding (Baldwin, 2014 ▸) and revisited Kauzmann’s theory of the critical role of the hydrophobic factor. Kauzmann explained how the hydrophobic factor works (Kauzmann, 1959 ▸), based on the proposal made by Frank and Evans that a hydrocarbon forms a hydration shell upon solvation in a water phase (Frank & Evans, 1945 ▸). The free energy of the hydration shell that is lost when hydrophobic side chains become buried in the core of the protein provided the missing free energy needed to rationalize protein folding.

Protein molecules are dynamic systems and interact with their environment. Frauenfelder and coworkers described the solvent surrounding a protein molecule as an independent moiety that contains a reservoir of entropy (Frauenfelder *et al.*, 2009 ▸). The protein surface provides donor/acceptor atoms that anchor a hydration shell. Protein crystallization was considered to be an entropy-driven phase transition rooted in the loss of free energy in the hydration shell owing to the migration of solvent molecules to the bulk solvent upon the assembly of the protein molecules into a crystal lattice (Vekilov *et al.*, 2002 ▸). Compared with protein folding, where the hydrocarbon is depleted of water in the core, in protein crystallization a small number of water molecules are lost from the hydration shell (Vekilov, 2003 ▸).

Protein crystals are self-assembled formations that differ from inorganic and organic crystals in the presence of space between the molecules that is filled with disordered solvent (Matthews, 1968 ▸). The void in the crystal and thermal motions in the bulk solvent are the factors that induce entropy in the protein crystal phase. In X-ray structural studies, this entropy has been described in terms of static and dynamic disorder (Burling & Brünger, 1994 ▸). Disordered amino-acid side chains or polypeptide segments are poorly resolved in electron-density maps and may require high-precision data for their visualization (Marquart *et al.*, 1980 ▸; Liebschner *et al.*, 2013 ▸).

Structural studies using NMR long ago showed the conformational heterogeneity of protein molecules in solution (for a review, see Montelione *et al.*, 2013 ▸). The subjects for which three-dimensional structures have been determined with high resolution are typically small proteins (<20 kDa); however, in some cases protein structures as large as 50 kDa have been determined with reasonable accuracy (Serrano, Aubol *et al.*, 2016 ▸; Serrano, Dutta *et al.*, 2016 ▸). NMR spectroscopy is used to characterize protein dynamics over a variety of time ranges from picoseconds to hours (Lange *et al.*, 2008 ▸; Kovermann *et al.*, 2016 ▸). On the other hand, the latter is a limitation of NMR because of the difficulty in quantifying dynamic motions that occur over wide ranges. To circumvent the latter limitation, Lindorff-Larsen and coworkers developed the dynamic ensemble refinement (DER) method and used it to produce a structural ensemble of ubiquitin starting from an X-ray structure, and cross-validated the ensemble using experimental data obtained by NMR (Lindorff-Larsen *et al.*, 2005 ▸).

Molecular-dynamics (MD) simulations have routinely been used to add a further dimension to the three-dimensional model of a protein molecule obtained with the use of X-ray diffraction methods: dynamics. MD simulation programs predict the movement of the individual atoms in a protein molecule and provide information about the dynamic behavior of the molecule that is needed to understand processes related to the mechanism of catalysis, protein–protein interactions, drug design and ligand binding. A recent refinement technique (Levin *et al.*, 2007 ▸) alleviates the compliance between observed and refined parameters by the use of an idea that underlies the unified model of protein dynamics (Frauenfelder *et al.*, 2009 ▸), which describes a protein molecule switching between a large number of conformational substates (CS). Ensemble refinement combines MD simulation with traditional crystallographic refinement. The resultant set of structures (the ensemble) provides more realistic information about the protein molecule by replacing the atomic fluctuations with multiple copies of the refined molecule (ensemble members). The unified model of protein dynamics postulates that a protein may assume a very large number of CS organ­ized in the energy landscape. For a polypeptide chain encompassing 100 residues, the number of CS is 10^100^. For this reason, no two molecules in solution adopt the same conformation at the same time, nor does a molecule return to the same conformation twice. Protein molecules switch between CS in finding a relevant (functional) conformation within lower energy level tiers called α-basins (Frauenfelder *et al.*, 2009 ▸).

There is a significant body of evidence in older data that examine the activity of crystalline enzymes (Doscher & Richards, 1963 ▸; Kallos, 1964 ▸; Sluyterman & de Graaf, 1969 ▸; Quiocho *et al.*, 1972 ▸; Honikel & Madsen, 1973 ▸; Kasvinsky & Madsen, 1976 ▸; Spilburg *et al.*, 1977 ▸). Although some of the enzymes may have lost a portion of their activity in the crystal, they still remained catalytically active. The latter observation suggests that the state of the protein in the crystal is similar to that in solution and that the unified model of protein dynamics (Frauenfelder *et al.*, 2009 ▸) should also be valid for the crystalline state of proteins. However, in another group of crystalline enzymes the catalytic activity was inhibited, possibly owing to restriction of the conformational flexibility of the particular enzyme owing to packing forces in the crystal. Recent publications have provided direct evidence that supports the notion of the validity of the universal model for the crystalline proteins. Wall *et al.* (2014 ▸) investigated the conformational dynamics of a crystalline protein with the use of accurately measured scattered X-ray diffraction obtained from protein crystals and used MD simulations to analyze the dynamics of the molecules in the crystal phase. They found eight metastable states of the structure of staphylococcal nuclease extending from 4 ns up to 1100 ns. Ma *et al.* (2015 ▸) observed rocking motions in the structure of ubiquitin crystallized in different morphologies. Using three techniques, X-ray diffraction, solid-state NMR and MD simulations, they found that the three-dimensional structure of ubiquitin oscillated on a time scale of 0.1 ms to 100 µs. The overall rocking motions in the crystal structure are likely to result in oscillating crystal contacts similar to those presented in Fig. 3 of the present study.

Ensemble refinement (Levin *et al.*, 2007 ▸) is less sensitive to the accuracy of the X-ray data compared with single-model refinement, and has been shown to improve the refinement statistics, therefore providing more detailed information about the biological macromolecule under investigation (Burnley *et al.*, 2012 ▸; Forneris *et al.*, 2014 ▸). Ensemble refinement has found relevant functional dynamics in the core of the protein. For instance, in three of the 20 selected proteins clusters of residues (up to 16) were found in multiple conformations that may facilitate the movement of ligands within the proteins (Burnley *et al.*, 2012 ▸). It is generally accepted that the protein core is a tightly packed and ordered matrix, although there is evidence that shows voids in the core (Katti *et al.*, 1989 ▸) as well as dynamic disorder (Hetzel *et al.*, 1976 ▸; Wagner *et al.*, 1976 ▸). According to recent data, dynamics in the core involving clusters of residues occur to such an extent that it is defined as a ‘molten core’; however, it is likely to be biologically relevant (Burnley *et al.*, 2012 ▸). In some proteins, for instance proline isomerase, the structure of which is available at ambient temperature (PDB entry 3k0n; Fraser *et al.*, 2009 ▸) and under cryogenic conditions (PDB entry 3k0m; Fraser *et al.*, 2009 ▸), there are differences in the dynamics in the core. While 11 side chains in the core form a dynamic cluster in the structure at ambient temperature, at cryogenic temperature they were well ordered, demonstrating that annealing of the conformations to the ground state has no biological meaning (Burnley *et al.*, 2012 ▸).

Little is known about the consequences of the dynamics at the surfaces of protein molecules refined using ensemble refinement. The present paper is intended to fill a gap in the literature regarding this aspect. Here, we report a dynamics *versus* time-averaged analysis of protein lattices, which shows that protein crystals are actually dynamic assemblies made up of molecules with elastic, oscillating shapes, effectively defining them as a material of their own class. They consist of two phases: one that is governed by the symmetry operator of the corresponding space group, and another that is found in a state that appears to be closer to the disordered bulk solvent owing to oscillations in the shape. The present observation expands the role of the shape entropy and local entropic forces (van Anders *et al.*, 2014 ▸) above the onset of crystallization. For the first time, the role of protein dynamics was extended beyond the asymmetric unit of protein crystals and was related to the symmetry of the crystals, which is a major determinant in crystal physics. A principle has also been formulated that is likely to determine the behavior of colloidal particles in a crowded environment. Our hypothesis is corroborated by the emerging data (Wall *et al.*, 2014 ▸; Ma *et al.*, 2015 ▸) and provides a platform for further experimental studies of the dynamics in protein crystal lattices.

## 2. Materials and methods   

The basic concept that underlies the present study is founded on the unified model of protein dynamics (Frauenfelder *et al.*, 2009 ▸). The void in the protein crystals is filled with disordered solvent (Matthews, 1968 ▸), which will generate entropy in the protein phase that will force the protein crystal lattice to oscillate in a correlated or noncorrelated mode (Devedjiev, 2015 ▸) in order to maintain crystal integrity.

The current study hypothesizes that the ‘molten’ state found in the core of proteins (Burnley *et al.*, 2012 ▸) is likely to also be found at the surface and may be involved in crystal contacts. For this reason, crystal contacts were analyzed within a set of ensemble members to ascertain whether or not all of the contacts were cohesive. The study investigated the occurrence of hydrogen bonds, van der Waals contacts and electrostatic interactions (in cases where the crystals were obtained at a pH that would suggest that the amino-acid chains of interest were ionized) and the lack of interactions between symmetry-related partners.

Wall *et al.* (2014 ▸) and Ma *et al.* (2015 ▸) observed, with the use of four independent techniques, that the main chains of protein molecules in crystals oscillate with a time range of 100 µs to 4 ns. If we presume that the side chains of these proteins oscillate in a similar range and take the lowest value of the oscillation rate, 100 µs, for a protein consisting of 100 amino-acid residues, the total number of oscillations for a molecule per second will be 100^5^. We constructed a hypothetical model crystal containing one molecule in the asymmetric unit surrounded by one symmetry-related neighbor in each crystallographic direction. The total number of independent oscillations for the hypothetical model crystal was 100^15 625^. Even accounting for some restrictions in the conformational degrees of freedom of the residues involved in crystal contacts, this number is meaningless and just vividly demonstrates that quantitative evaluation of the conformational entropy on a microscopic level in protein crystals is not a subject of scientific interest. For this reason, as in our previous study (Devedjiev, 2015 ▸), we used statistical approaches to evaluate the conformational entropy in protein crystal lattices.

Quantitative evaluation of the entropy of protein crystallization on a macroscopic level has been performed before (Vekilov, 2003 ▸), and it was experimentally determined that entropy is the driving force in protein crystal formation. Attempts have been made to determine the role of conformational entropy on a microscopic level and to relate it to the physical properties of the amino acids involved in crystal contacts (Derewenda, 2004 ▸; Derewenda & Vekilov, 2006 ▸; Cieślik & Derewenda, 2009 ▸). These studies used a formalism that included coefficients for the side-chain conformational entropy (SCE coefficients) of individual amino-acid side chains that were calculated for uniform conditions: protein folding. SCE coefficients are not applicable to protein crystallization, which occurs under non-uniform conditions (Devedjiev, 2015 ▸), and not surprisingly the results of these studies did not correlate with those obtained by direct observation (Devedjiev, 2015 ▸; Dasgupta *et al.*, 1997 ▸). To circumvent the limitations noted above, and to evaluate conformational entropy on a microscopic level, we constructed a qualitative physical model that defined the protein molecules in the asymmetric unit as elastic entities with oscillating shapes, caged in the lattice by the packing forces. Cage walls were described as a contact area between symmetry-related molecules along the corresponding crystallographic axis. To assess the spatial distribution of the conformational entropy, two types of cage were analyzed: anisotropic (space group *P*2_1_) and isotropic (space group *P*2_1_2_1_2_1_). We calculated all symmetry-related molecules using *CONTACT* (Winn *et al.*, 2011 ▸) and investigated the population of relevant crystallographic directions (cage walls) with amino-acid side chains for a selection of ensemble members. The solvent-accessible surface area (SASA) was also calculated for the ensemble members of interest as well as the SASA in the crystal contacts.

20 protein structures refined with ensemble refinement were downloaded from http://datadryad.org/resource/doi:10.5061/dryad.5n01h and encompassed the working data set of Burnley *et al.* (2012). Stereochemical analysis of the protein structures and symmetry-related partners was performed with *Coot* (Emsley & Cowtan, 2004 ▸). Contacts between symmetry-related molecules were analyzed using distance cutoffs of a minimum of 2.2 Å and a maximum of 5.0 Å. The SASA was calculated with a probe radius of 1.4 Å, and a minimum cutoff of 2.0 Å^2^ for a solvent-exposed residue was applied. The cutoff applied for electrostatic interactions between charged side chains was 6 Å. All crystallographic computations were performed with *CCP*4 (Winn *et al.*, 2011 ▸). Illustrations were prepared with *PyMOL* (DeLano, 2002 ▸) and *Coot* (Emsley & Cowtan, 2004 ▸).

## 3. Results and discussion   

Recent findings (Devedjiev, 2015 ▸) have demonstrated that static disorder and conformational entropy are tolerated by protein crystal lattices despite the views noted in existing theory (Derewenda, 2004 ▸; Derewenda & Vekilov, 2006 ▸). To further assess the role of conformational entropy in protein crystallization, protein structures refined using the ensemble-refinement technique were analyzed (see §[Sec sec2]2).

### 3.1. Evidence that establishes conformational entropy as a major factor for the emergence and integrity of the protein condensed phase   

The data given in Tables 1 ▸ and 2 ▸, in which the solvent-accessible surface area (SASA) was calculated for the 39 available ensemble members in the structure of a transcriptional antiterminator (PDB entry 3gwh; Rodríguez *et al.*, 2009 ▸) and for the first 50 ensemble members, out of a total of 600, in the structure of HIV hydrolase (PDB entry 1kzk; Reiling *et al.*, 2002 ▸), show a random distribution of a variable that quantitatively represents the shape of the proteins. Differences in the SASA in the crystal contacts of the selection of ensemble members studied for 3gwh reached 11%, whereas the changes in the total SASA were 13%. In HIV protease, the differences were even greater: 17% for the SASA in crystal contacts and 16% for the total SASA. These markedly large differences were suggestive that some ensemble members may not form crystal contacts in particular intermolecular areas.

**Figure 1:**
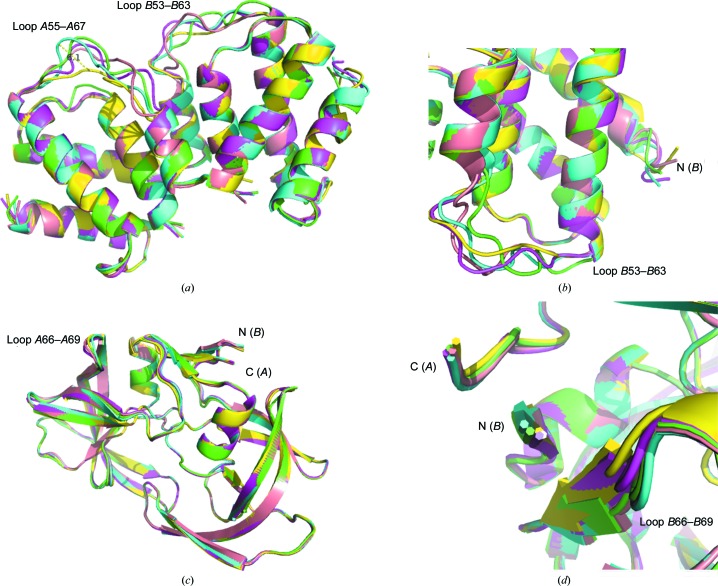
Rigid-body dynamics in the main-chain atoms of protein structures refined using the ensemble-refinement technique. (*a*) Five ensemble members in the structure of the transcriptional antiterminator (PDB entry 3wgh) display conformational flexibility of two flexible loops enabled by the symmetry of the lattice. (*b*) Flexibility at the N-terminus of molecule *B*. Ensemble members are color-coded as follows: 1, green; 2, blue; 3, lavender; 5, yellow; 8, pink. (*c*) Five ensemble members in the structure of HIV hydrolase (PDB entry 1kzk) show a lack of significant conformational flexibility in the flexible loops and termini because of the three orthogonal twofold screw axes that define the symmetry operator and restrict the degrees of freedom. (*d*) The N- and C-­termini of HIV protease do not display flexibility. The coloring scheme is as follows: ensemble member 9, green; ensemble member 18, blue; ensemble member 26, lavender; ensemble member 27, yellow; ensemble member 32, pink. The distance between C^α^ atoms of ensemble members is also shown.

In a polar cage, one would expect degrees of freedom along the twofold screw axis, because if there were symmetry operators perpendicular to the polar axis they would provide an identical environment in all crystallographic directions. Indeed, the flexible loops *A*55–*A*67 and *B*53–*B*63 and the termini explore degrees of freedom as a rigid body (Figs. 1 ▸ *a* and 1 ▸ *b* and Supplementary Fig. S1). However, in a nonpolar cage, where the three orthogonal screw axes provide an identical crystallographic environment along all three of the crystallo­graphic axes, the flexible loops *A*66–*A*69 and *B*66–*B*69 and the termini are restricted in movement (Figs. 1 ▸ *c* and 1 ▸ *d*). Furthermore, analysis of the entropy in five randomly selected ensemble members from both molecules did not find any role for the symmetry operator in the intermolecular contacts (Tables 3 ▸ and 4 ▸). The differences in the populations of amino-acid side chains in the cage walls in the 010 and 0−10 directions can be explained by the symmetry of the space group *P*2_1_ that allows degrees of freedom along the polar axis (see §[Sec sec2]2). However, the differences in the populations of amino-acid side chains in the 100, −100 and 001, 00−1 directions do not follow the law of symmetry inferred by the symmetry operator for the *P*2_1_ space group, which requires an equal population of amino-acid side chains in the cage walls in symmetrically related directions (Supplementary Table S3).

**Figure 2:**
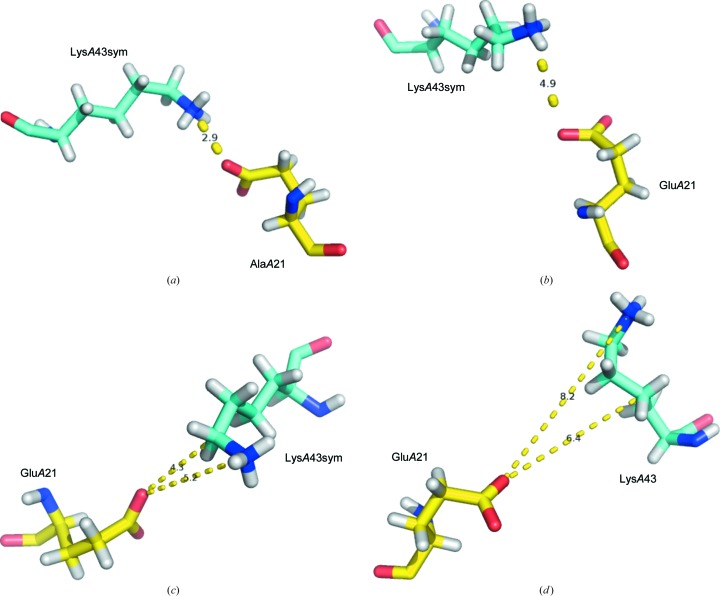
The stochastic nature of protein crystal contacts. (*a*) A salt bridge in ensemble member 90 in the structure of HIV hydrolase between Glu*A*21 of the reference molecule and Lys*A*43 of the symmetry-related molecule forms a cohesive interaction that is part of the crystal lattice. (*b*) In ensemble member 40 in the structure of HIV hydrolase, Glu*A*21 of the reference molecule and Lys*A*43 of the symmetry-related molecule contact by means of electrostatic interaction. (*c*) Salt bridge and van der Waals interactions in ensemble member 28. (*d*) In ensemble member 1 in the structure of HIV hydrolase Glu*A*21 and Lys*A*43sym are not located at a proximity to form a lattice contact.

**Figure 3:**
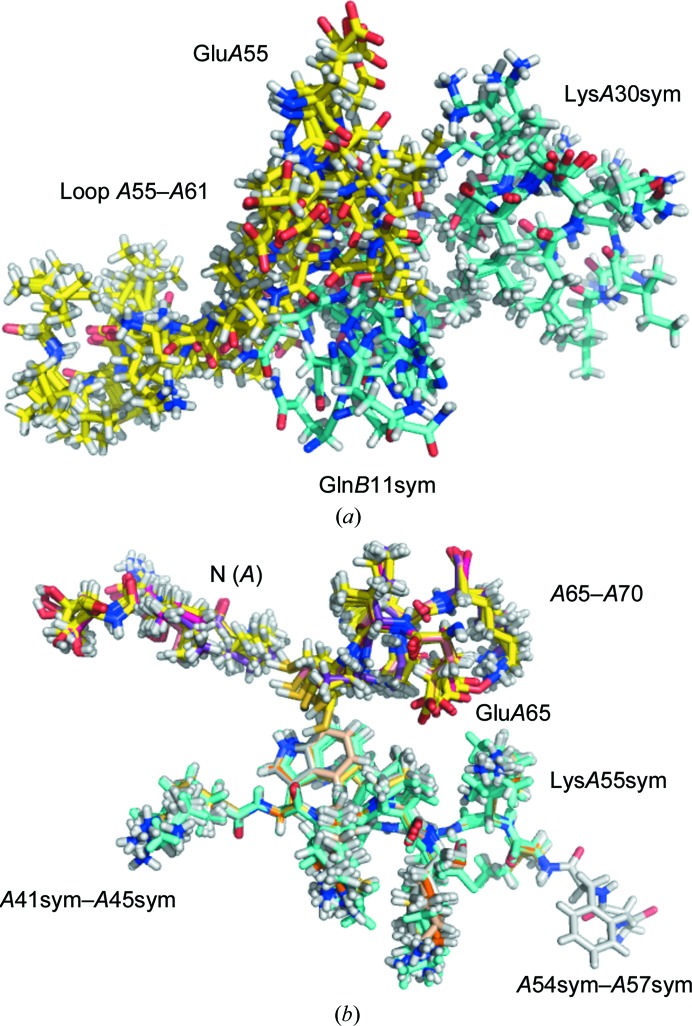
Lattice dynamics in protein crystals. (*a*) Polar cage: a crystal contact in the structure of the transcriptional antiterminator and its symmetry-related partner. (*b*) Nonpolar cage: a crystal contact in the ensemble in the structure of HIV hydrolase. Ten ensemble members are presented in both cases. Reference molecules are shown with yellow bonds and symmetrically related molecules with cyan bonds.

A snapshot encompassing all 600 ensembles in the structure of HIV hydrolase (Supplementary Fig. S2) shows that some of the ensemble members are too distant to interact with a symmetry-related molecule. In Figs. 2 ▸(*a*), 2 ▸(*b*), 2 ▸(*c*) and 2 ▸(*d*) the oscillations of a particular symmetry-related pair of residues demonstrate that the pair is found in four states: forming a salt bridge (Fig. 2 ▸ *a*), forming an electrostatic interaction (Fig. 2 ▸ *b*), making an electrostatic interaction and a van der Waals contact (Fig. 2 ▸ *c*) and, finally, with a lack of contact (Fig. 2 ▸ *d*). Crystal contacts were compared in pairs of ensemble members that represent the structures of HIV protease and the transcriptional antiterminator (Figs. 3 ▸ *a* and 3 ▸ *b*), and a random distribution of cohesive and noncohesive constellations between side chains in the contact area was found. Here, we argue that the terms ‘contact residue’ and ‘contact area’ in protein crystal lattices are relative by nature owing to the flexibility of the surface and are dynamic. A time-averaged crystal structure may not identify all possible contact residues. Fig. 3 ▸(*a*) and Supplementary Figs. S2, S3 and S4 vividly demonstrate a range of oscillations of a dynamic protein model that go far beyond static disorder in the time-averaged models. The data for the ranges of disagreement between the time-averaged model and selected ensemble members are presented in Table 5 ▸. We reached the conclusion that any surface-exposed residue within ensemble members with a potential to form an interaction, based on conformational degrees of freedom, with a symmetry-related molecule at any time could be defined as a contact residue. The data presented above clearly show that conformational entropy only increases the probability of a contact event occurring, leading to a cohesive interaction that would involve a hydrogen bond, van der Waals or electrostatic interaction (enthalpic factor). The known theory for reduction of the surface entropy in the crystallization of proteins relates the conformational entropy of a protein molecule to its crystallization propensity (Derewenda, 2004 ▸). The conformational entropy of a protein molecule is a microscopic parameter and a property of an individual particle, and is not related by a physical law to the entropy of crystallization, which is a macroscopic parameter and a property of the system (Derewenda & Vekilov, 2006 ▸). These are parameters of incompatible ranks. In protein crystallization, entropy accounts only for the change in the number of particles in the ordered phase (crystal) and dis­ordered phase (solute) and disregards individual properties of the particles. On one protein molecule incorporated into the crystal lattice, many water molecules will leave the ordered hydration shell and will join the disordered bulk solvent (Vekilov *et al.*, 2002 ▸); therefore, the entropy of the system will increase and the free energy will decrease. In contrast to the current theory (Derewenda, 2004 ▸), the present study demonstrates that conformational entropy of a protein molecule appears to be the major factor that supports the formation of the protein condensed phase and works in synergy with enthalpy to maintain the integrity of the phase.

### 3.2. Evidence that establishes local entropic forces as a general factor that determines the behavior of colloidal particles in a crowded environment   

It has previously been found (van Anders *et al.*, 2014 ▸) that shape entropy is a driving force in local dense packing just below the onset of crystallization. The present study compared the occurrence of surface entropy in areas of crystal contacts with the analogous surface entropy in areas of the molecule that were not involved in contacts. The results given in Tables 6 ▸ and 7 ▸ show increased local entropy in the contact area in the polar lattice. The flexibility in the contact area was 2.14 times larger compared with noncontact areas of the molecules (Supplementary Tables S1 and S2; maximum deviation of 12.83 Å). In a nonpolar cage the corresponding value was 1.67 times, with a maximum deviation of 6.49 Å (Tables 7 ▸ and 8 ▸; Supplementary Tables S3 and S4), which is consistent with the increased degrees of freedom for side chains in a polar cage. Interestingly, in a polar cage the secondary structure shows significant flexibility in the contact area, where it is 5.11 times larger, whereas in a nonpolar cage it is only 1.27 times larger.

The data presented in the current study were inferred from 20 structures refined with ensemble refinement, and those reported in our previous studies based on the occurrence of conformational flexibility (surface entropy) in crystal contacts of 105 protein structures refined at high resolution (Devedjiev, 2015 ▸). The data expand the role of shape entropy as a driving force in the formation of a colloidal condensed phase and maintaining its integrity above the onset of crystallization. Therefore, they provide solid evidence that covers the full range of experimental conditions that generate and maintain the integrity of self-assembled colloidal formations. We suggest that owing to the general character of this principle, it could be formulated as follows: *in self-assembled systems, in crowded environments, containing colloidal particles dissolved in a liquid phase, local entropy is maximized in the contacts between the particles*. This can also be presented as the formula
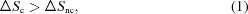
where Δ*S*_c_ is the change in conformational entropy on transition from the liquid to the crystalline phase in areas involved in crystal contacts and Δ*S*_nc_ is the change in the conformational entropy in areas that were not involved in crystal contacts. Here, we need to point out that this principle correlates to the principle formulated by Wukovitz & Yeates (1995 ▸) for the minimum number of unique contacts in the protein crystal lattices that relates to more degrees of freedom for a protein molecule in a crystal.

The unusual, but logical, consequence of this principle, when strictly considered, is that protein crystals do not fall into the definition of the solid state of matter: they are a material of their own class. We need to note that the formula shown above does not represent strict quantitative relationships; it is not an equation. It just compares two quantities in the sense of probability of occurrence. A higher probability of occurrence of conformational entropy was found in crystal contacts that provided grounds to expand the occurrence of entropy in the condensed phase to the general case.

### 3.3. Ensembles represent real-time oscillations of the protein structure at ambient temperature   

It has been known since the early days of crystallography that protein crystals represent two phases: an ordered protein phase governed by a symmetry operator and a disordered solvent phase that fills the void between molecules in the lattice. The bulk solvent contains a reservoir of entropy and drives entropy in the hydration shell that surrounds the protein (Frauenfelder *et al.*, 2009 ▸). The protein hydration shell fluctuates on its own timescale compared with the protein, with differences ranging from the picosecond to the nano­second timescale (Nickels *et al.*, 2012 ▸). Figs. 1 ▸(*a*), 1 ▸(*b*), 1 ▸(*c*) and 1 ▸(*d*) present oscillations in the secondary structure that can be rationalized as a rigid-body movement described by the symmetry operator. The ensemble members that are shown in Supplementary Fig. S2 and in Figs. 2 ▸(*a*), 2 ▸(*b*) and 2 ▸(*c*) are traditionally believed to be different conformations of the side chains within the volume of the crystals, characterized quantitatively by an occupancy factor. In studies that report the structures of the transcriptional antiterminator and HIV hydrolase this is exactly the case because the data were collected at cryogenic temperatures from crystals in a vitreous state. At ambient temperature, the conformations presented in Figs. 2 ▸(*a*), 2 ▸(*b*) and 2 ▸(*c*) are states governed by the symmetry operator because they occur as a result of interaction with the ordered phase. The conformations shown in Fig. 2 ▸(*d*) and some of those in Supplementary Fig. S2, however, are transient because they are in contact with the dynamic phases (the hydration shell or the bulk solvent) and will explore the conformational space on a timescale that is experimentally measurable with the use of advanced structural methods based on X-ray free-electron lasers. Wall *et al.* (2014 ▸) and Ma *et al.* (2015 ▸) determined the oscillations of main-chain atoms in the microsecond to picosecond range, which is within the reach of the abovementioned methods. Analysis of the crystal contacts in 20 structures refined with ensemble refinement (Burnley *et al.*, 2012 ▸) shows that dynamics in the lattices are a common feature of protein crystals.

### 3.4. van der Waals interactions dominate in the formation of protein crystal lattices   

Figs. 2 ▸(*a*), 2 ▸(*b*) and 2 ▸(*c*) demonstrate that all types of interactions that are known to exist in protein molecules also occur in the crystal contacts. We took this one step further and quantified the occurrence of interactions by type in the crystal lattices of two protein molecules: one with experimentally determined positions of H atoms (PDB entry 3a38; Takeda *et al.*, 2010 ▸) and another with theoretically predicted positions (PDB entry 1kmt; Mateja *et al.*, 2002 ▸). The results are shown in Table 8 ▸. Astonishingly, 97% of the contacts are owing to hydrogen-mediated crystal contacts. These results reveal a never before considered role of H atoms in crystalline proteins. ‘Stripping’ protein molecules of H atoms which are present their native form provides an artificially elevated role for hydrogen bonds and electrostatic interactions and masks the real interrelationship. We call this an ‘octopus effect’. When moving on the surface of a coral, an octopus uses its arms, which are covered with suction cups or suckers. The suction cups located on the lower surface of the arm are so numerous that even when part of the arm detaches from the surface of the coral, the suction cups of the remaining part will still keep the body of the octopus firmly attached to the coral. When oscillating in the crystal lattice, even when a particular side chain(s) loses contact with the symmetry-related molecule (Figs. 2 ▸ *d* and 3 ▸, Supplementary Figs. S3 and S4) there are still significant numbers of interactions to maintain the integrity of the protein crystal lattice (Table 8 ▸). This explains the dominant role of van der Waals interactions in the formation of protein crystal lattices, a notion that has never before been recognized.

### 3.5. Ensemble refinement confirms that the time-averaged model is not the most accurate representation of the protein structure in solution   

Lindorff-Larsen *et al.* (2005 ▸) compared a single static structure with an ensemble of structures that were constrained to yield a simultaneous fit with two different groups of NMR parameters: structural (NOE, *J* coupling and RDCs) and dynamic (^15^N relaxation rates). The comparison showed that the ensemble yields better cross-validation statistics.

It is well known that dynamic disorder is not within the reach of single-model refinement. Liebschner *et al.* (2013 ▸) first quantitatively evaluated the reproducibility of time-averaged models and found that the conventional refinement technique cannot successfully model flexible parts of the protein structure even at atomic resolution (0.75 Å). Traditional methods of X-ray data collection rely on the physics of X-ray diffraction, which is based on the interaction of a photon with an electron that circulates around the nucleus in an unpredictable manner and is stochastic (Johnson & Blundell, 1976 ▸). For this reason, the acquisition of data with reasonable statistics may require times of at least minutes at third-generation synchrotrons or more at the home source. During the length of data collection, a protein molecule in the crystal may have adopted a significant number of conformations that are experimentally measurable. In ensemble refinement, Levin and coworkers used MD to model the dynamic disorder and demonstrated that ensemble refinement is consistent in improving the disagreement between crystallographic and *R*_free_ factors independent of the accuracy of the data (Levin *et al.*, 2007 ▸). Further studies improved the algorithms for the ensemble refinement and found melts in the core of the protein as presented by the refined ensemble (Burnley *et al.*, 2012 ▸). Tables 5 ▸, 6 ▸ and 7 ▸ give a quantitative illustration of the scale of the conformational changes occurring in the crystal lattices of ensemble-refined proteins. The differences in the positions of the atoms within the ensemble that are identical chemical entities are larger than the typical deviation from homologous proteins derived from different species with a sequence similarity that is much lower that 100%. Keeping in mind that conformational degrees of freedom in the crystal are restricted by the type of lattice (Supplementary Figs. S3 and S4), in solution one could expect even larger deviations from the time-averaged model than those reported in Table 5 ▸.

## 4. Conclusions   

We show here, through the analysis of conformational entropy in two protein crystal lattices that belong to polar and non­polar space groups, that the intermolecular contacts in the protein condensed phase are dynamic by nature.

Based on 20 ensemble-refined structures and corroborated by 105 structures that have been analyzed by us before, we have shown that the role of shape entropy and local entropic forces expands above the onset of crystallization, and we have formulated a principle that determines the behavior of colloidal particles in a crowded environment.

Static disorder is defined in the time-averaged models as the distribution of side-chain conformations within the volume of the protein crystal determined by the occupancy factor. Actually, at ambient temperature, it represents the ensemble of conformational states.

Analysis of the intermolecular interactions in the crystals of two native protein molecules containing experimentally determined H atoms or theoretically assigned H atoms vividly demonstrates that hydrogen-mediated van der Waals interactions are the dominant force that maintains the integrity of protein crystal lattices. ‘Stripping’ protein molecules of H atoms leads to an artificially elevated role for hydrogen bonds and electrostatic interactions and masks the real interrelationships in the protein contact area.

## Supplementary Material

Supplementary Figures S1 and S2.. DOI: 10.1107/S2052252517017833/mf5019sup1.pdf

## Figures and Tables

**Table 1:** Surface dynamics in an anisotropic cage (space group *P*2_1_) The SASA is given for 39 ensembles in the structure of the transcriptional antiterminator (PDB entry 3wgh; for details, see the text). The shape of the molecules is quantitatively represented by the size of the SASA.

	SASA in contact	Total SASA
Minimum value of SASA (Å^2^)	1774	6002
Maximum value of SASA (Å^2^)	1985	6934
Difference (%)	11	13

**Table 2:** Surface dynamics in an isotropic cage (space group *P*2_1_2_1_2_1_) 600 ensembles in the structure of HIV protease refined to 1.09 Å resolution (PDB entry 1kzk; for details, see the text). The shape of the molecules is quantitatively represented by the size of the SASA.

	SASA in contact	Total SASA
Minimum value of SASA (Å^2^)	1513	5133
Maximum value of SASA (Å^2^)	1819	6109
Difference (%)	17	16

**Table 3:** Surface dynamics in an anisotropic cage in a randomly selected selection of ensembles (space group *P*2_1_; PDB entry 3gwh) For details, see the text. Ref, reference molecule. Sym, symmetry-related molecule.

	Cage walls							
Ensemble No.	100	−100	010	0−10	001	00−1	SASA in contacts	Total SASA
1								
No. of contact residues	40	72	0	23	20	69		
SASA in cage wall (Sym/Ref) (Å^2^)	4036/4012	6995/7671	0	2222/2585	1947/1867	7564/6646	3853	13043
2								
No. of contact residues	28	0	15	15	24	32		
SASA in cage wall (Sym/Ref) (Å^2^)	2630/3129	0	1532/2006	1411/1646	2398/2066	3358/3283	3832	12960
3								
No. of contact residues	31	65	0	24	28	58		
SASA in cage wall (Sym/Ref) (Å^2^)	3111/3281	7016/6887	0	2162/3026	3033/2773	5965/5751	3696	12516
5								
No. of contact residues	32	61	0	15	25	35		
SASA in cage wall (Sym/Ref) (Å^2^)	3430/4041	6984/7123	0	1879/1557	3173/3685	4723/5218	3662	12409
8								
No. of contact residues	43	74	0	20	25	60		
SASA in cage wall (Sym/Ref) (Å^2^)	4204/4479	7529/8047	0	2301/2674	2365/2518	6216/5462	3804	12860
Minimum (Å^2^)							3662	12409
Maximum (Å^2^)							3853	13043
Minimum (Ref/Sym) (Å^2^)	2630/3129	6984/7671	0/0	1411/1557	1947/1867	3358/3283		
%	16	9		9	6	2		
Maximum (Ref/Sym) (Å^2^)	4036/4479	7529/8047	1532/2006	2301/3026	3173/3685	7564/6645		
%	10	6		24	14	12		

**Table 4:** Surface dynamics in an isotropic cage in a randomly selected selection of ensembles (space group *P*2_1_2_1_2_1_; PDB entry 1kzk) For details, see the text. Ref, reference molecule. Sym, symmetry-related molecule.

	Cage walls							
Ensemble No.	100	−100	010	0−10	001	00−1	SASA in contacts	Total SASA
9								
No. of contact residues	64	24	0	7	0	18		
SASA in cage wall (Sym/Ref) (Å^2^)	7179/6519	2073/2383	0	825/899	0	1567/2166	3498	11809
18								
No. of contact residues	58	28	0	9	0	19		
SASA in cage wall (Sym/Ref) (Å^2^)	6102/5585	2278/2355	0	948/1156	0	1956/2261	3493	11827
26								
No. of contact residues	57	26	0	7	0	21		
SASA in cage wall (Sym/Ref) (Å^2^)	5997/5150	2117/2289	0	870/793	0	1968/2277	3155	
27								
No. of contact residues	59	27	0	7	0	18		
SASA in cage wall (Sym/Ref) (Å^2^)	6197/5266	2126/2290	0	830/849	0	1519/2057	3147	10291
32								
No. of contact residues	64	26	0	7	0	19		
SASA in cage wall (Sym/Ref) (Å^2^)	5727/5110	2259/2222	0	878/840	0	1894/1698	3146	10630
Minimum (Å^2^)	57	24	0	7	0	18		
Maximum (Å^2^)	64	28	0	9	0	21		
Minimum (Ref/Sym) (Å^2^)	5727/5110	2073/2222	0	825/793	0	1519/1698	3146	10291
%	11	7		4		10		
Maximum (Ref/Sym) (Å^2^)	7179/6519	2278/2355	0	948/1156	0	1968/2277	3498	11809
%	9	3		18		14		

**Table 5:** Comparison between the time-averaged model and selected ensemble members in the transcription antiregulator All distances are given in Å.

Ensemble	1	2	3	5	8
C^α^	1.49	1.36	1.37	1.20	1.53
All atoms	1.89	1.85	1.78	1.81	1.91
C^α^ in loops *A*55–*A*67 and *B*53–*B*63	3.37	3.37	3.15	2.72	3.50
All atoms in loops *A*55–*A*67 and *B*53–*B*63	3.94	3.76	3.83	3.52	3.92

**Table 6:** Shape entropy in the protein condensed phase: polar cage The occurrence of shape entropy is determined by the fluctuation of 49 surface-exposed amino-acid residues in the crystal contacts of the transcriptional antiterminator (PDB entry 3gwh). Differences between C^α^ atoms and the terminal amino-acid residues are shown as a measure of surface conformational entropy. Some amino-acid side chains of residues located at the edges of the asymmetric unit switch directions, occupy neighboring cage walls within the ensemble or lose contact with the symmetry-related molecule.

Ensemble member No.	Differences in C^α^ atoms (Å)	Differences in terminal side-chain atoms (Å)	Occurrence of side chains in cage walls
1	0.98	1.20	−100, −1−10, 001, 00−1, −10−1, 100, 0−10, 0−1−1
2	0.90	1.81	−100, −1−10, 001, −10−1, 00−1, 100, 0−10, 0−1−1
3	0.81	1.36	−100, −1−10, 001, 00−1, −10−1, 100, 0−10, 0−1−1
4	1.03	1.50	−100, −1−10, 001, −10−1, 00−1, 100, 0−10, 0−1−1
5	0.73	1.38	−100, −1−10, 001, 00−1, −10−1, 100, 0−10, 0−1−1
6	0.75	1.25	−100, −1−10, 001, 00−1, −10−1, 100, 0−10, 0−1−1
7	0.79	1.37	−100, −1−10, 001, −10−1, 00−1, 100, 0−10, 001, 0−1−1
8	1.08	1.51	−100, −1−10, 001, −10−1, 100, 0−10, 0−1−1, 00−1
9	0.87	1.52	−100, −1−10, 001,−10−1, 00−1, 100, 0−10, 0−1−1, −10−1
10	0.84	1.76	−100, −1−10, 001, 00−1, −10−1, 100, 0−10, −10−1, 0−1−1
Minimum	0.73	1.20	
Maximum	1.08	1.81	

**Table 7:** Shape entropy in the protein condensed phase: nonpolar cage The occurrence of shape entropy is determined by the fluctuation of 36 surface-exposed amino-acid residues in the crystal contacts of HIV protease (PDB entry 1kzk). Differences between C^α^ atoms and the terminal amino-acid residues are shown as a measure of surface conformational entropy. Some amino-acid side chains of residues located at the edges of the asymmetric unit switch directions, occupy neighboring cage walls within the ensemble or lose contact with the symmetry-related molecule.

Ensemble member No.	Differences in C^α^ atoms (Å)	Differences in terminal side-chain atoms (Å)	Occurrence of side chains in cage walls
1	0.05	0.84	00−1, 100, 10−1, −100, 0−10, 1−10
2	0.05	1.00	00−1, 100, 10−1, −100, 0−10, 1−10
3	0.12	0.92	00−1, 100, 10−1, −100, 0−10, 1−10
4	0.19	0.73	00−1, 100, 10−1, −100, 0−10, 1−10
5	0.09	0.67	00−1, 100, 10−1, −100, 0−10, 1−10
6	0.14	0.78	00−1, 100, 10−1, −100, 0−10, 1−10
7	0.13	0.65	00−1, 100, 10−1, −100, 0−10, 1−10
8	0.13	0.67	00−1, 100, 10−1, −100, 0−10, 1−10
9	0.18	0.82	00−1, 100, 10−1, −100, 0−10, 1−10
10	0.17	0.78	00−1, 100, 10−1, −100, 0−10, 1−10
Minimum	0.05	0.65	
Maximum	0.19	1.00	

**Table 8:** The occurrence of intermolecular interactions by type in the crystal lattices of two proteins, high-potential iron–sulfur protein (PDB entry 3a38) and RhoGDI inhibitor (PDB entry 1kmt), demonstrates the insignificance of hydrogen bonds and electrostatic interactions in native (with H atoms) proteins in crystal lattices

		Hydrogen bonds		Electrostatic		van der Waals, non-H atoms		van der Waals, H-atom mediated	
PDB entry	Total No. of interactions	No.	%	No.	%	No.	%	No.	%
3a38	6142	21	0.3	5	0.09	157	3	5995	97
3a38 −H	183	21	11	5	3	157	86		
1kmt	5908	34	0.6	3	0.05	117	2	5764	97
1kmt −H	144	24	17	3	2	117	81		
