# Supplementary material for: Protein crystal lattices are dynamic assemblies: the role of conformational entropy in the protein condensed phase
Source: IUCrJ. 2018 Jan 10;5(Pt 2):130–40. doi: 10.1107/S2052252517017833 (PMC5947717; doi:10.1107/S2052252517017833)
Supplement: Supplementary file 1 [file m-05-00130-sup1.pdf]

IUCrJ

Volume 5 (2018)

Supporting information for article:

Protein crystal lattices are dynamic assemblies: the role of conformational entropy in the protein condensed phase

Margarita Dimova and Yancho D. Devedjiev

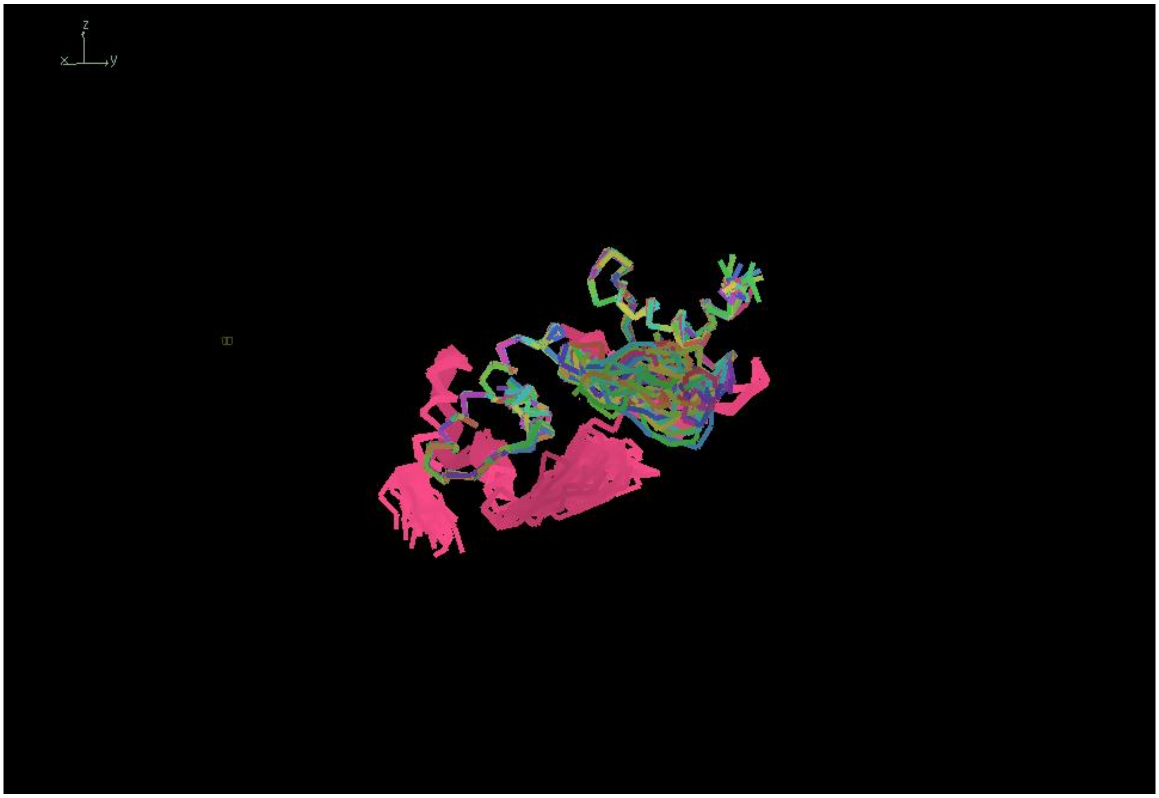

Figure S1 Flexible loops A55-A67; B53-B63 and the termini explore degrees of freedom as a rigid body.

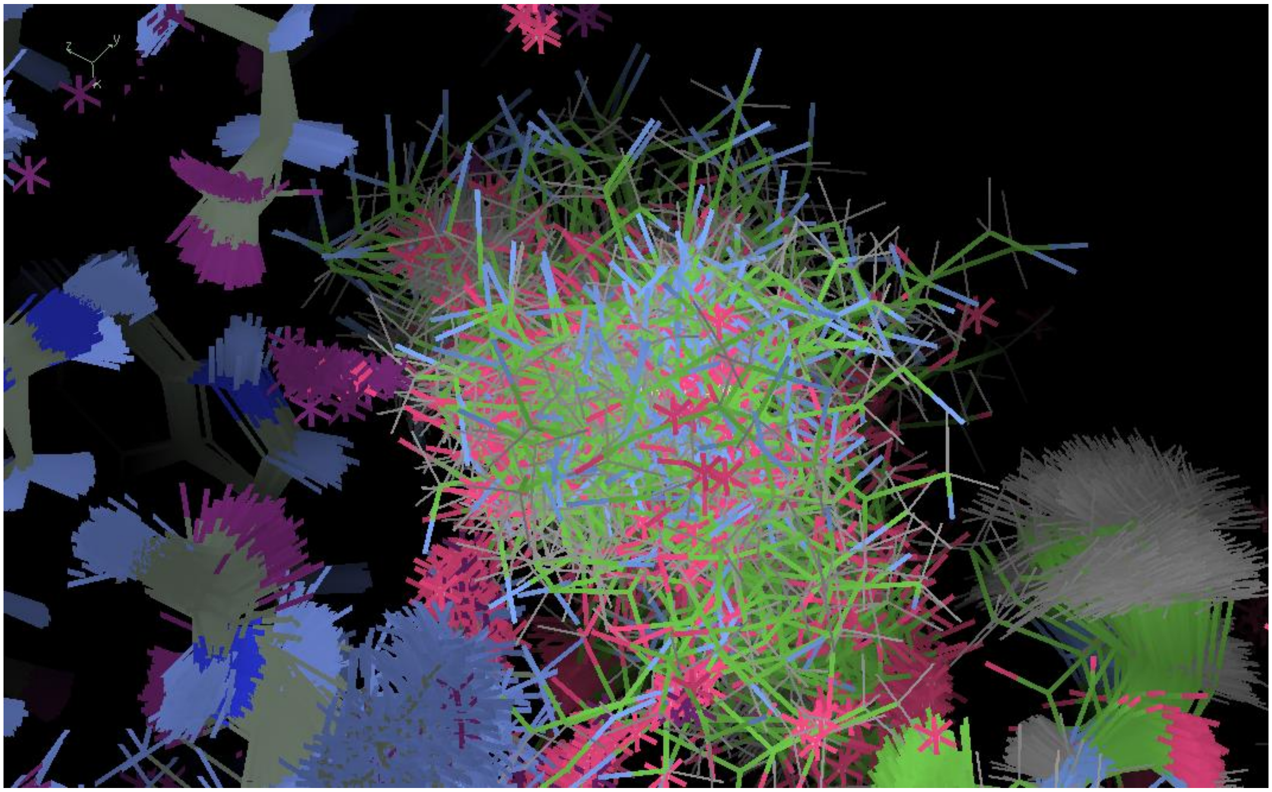

Figure S2 A snapshot encompassing all six hundred ensembles in the structure of HIV hydrolase.
